# The 3.5-year to 13.5-year follow up of 137 lesions of uncertain malignant potential (B3 lesions) diagnosed by vacuum-assisted biopsy alone

**DOI:** 10.1186/bcr3790

**Published:** 2015-11-05

**Authors:** Karen Mullin, Anne Turnbull, Sharma Puri, Mark Bagnall

**Affiliations:** 1 Royal Derby Hospital, Derby, UK

## Introduction

Vacuum-assisted biopsy (VAB) was introduced in Derby in 2001, as the second procedure after 14g core biopsy. We present 3.5-year to 13.5-year follow up of cases where B3 lesions have been managed with VAB alone.

## Methods

The NBSS and local BASO databases were searched from January 2002 to December 2011 for all cases with B3 histopathology, a VAB procedure and no surgery. Screening and symptomatic women were included.

## Results

There were 137 women who met the criteria. The pathologies found are presented in Table 1. The cases where atypia was found were individually discussed at MDT to ensure that the abnormal feature had either been excised or very well sampled. Only one breast cancer has developed at the same site in a woman who had 5 mm calcification excised at VAB. This lobular cancer was identified 4 years later at recall from annual surveillance. Five other cancers have developed in the 137 cases, one contralaterally and four different lesions in different sites in the same breast.

**Table 1:** 

Pathology	Initial biopsy	Final pathology
CSL	39	37
Papillary lesion, no atypia	51	50
Papillary lesion, atypia	2	0
CCC atypia/flat epithelial atypia	12	0
ADH/apocrine atypia/AIDEP	9	1
Lobular neoplasia	6	5
Mucocoele-like lesion	5	0
Other B3 atypia	5	0
Fibroepithelial lesion	2	0
B2	4	40
Fibroadenoma	0	3
B1	2	0
VAB only	0	1
Total	137	137

## Conclusion

This study provides further evidence for the safety of the use of VAB alone in the diagnosis of B3 lesions in the longer term.

